# Circulating Tumor Cells Count and Morphological Features in Breast, Colorectal and Prostate Cancer

**DOI:** 10.1371/journal.pone.0067148

**Published:** 2013-06-27

**Authors:** Sjoerd T. Ligthart, Frank A. W. Coumans, Francois-Clement Bidard, Lieke H. J. Simkens, Cornelis J. A. Punt, Marco R. de Groot, Gerhardt Attard, Johann S. de Bono, Jean-Yves Pierga, Leon W. M. M. Terstappen

**Affiliations:** 1 Department of Medical Cell BioPhysics, MIRA Institute, University of Twente, Enschede, The Netherlands; 2 Department of Medical Oncology, Institut Curie, Paris, France; 3 Department of Medical Oncology, Academic Medical Center, University of Amsterdam, Amsterdam, The Netherlands; 4 Department of Internal Medicine, Medisch Spectrum Twente, Enschede, The Netherlands; 5 The Royal Marsden NHS Foundation Trust, Sutton, Surrey, United Kingdom; National Cancer Center, Japan

## Abstract

**Background:**

Presence of circulating tumor cells (CTC) in patients with metastatic breast, colorectal and prostate cancer is indicative for poor prognosis. An automated CTC (aCTC) algorithm developed previously to eliminate the variability in manual counting of CTC (mCTC) was used to extract morphological features. Here we validated the aCTC algorithm on CTC images from prostate, breast and colorectal cancer patients and investigated the role of quantitative morphological parameters.

**Methodology:**

Stored images of samples from patients with prostate, breast and colorectal cancer, healthy controls, benign breast and colorectal tumors were obtained using the CellSearch system. Images were analyzed for the presence of aCTC and their morphological parameters measured and correlated with survival.

**Results:**

Overall survival hazard ratio was not significantly different for aCTC and mCTC. The number of CTC correlated strongest with survival, whereas CTC size, roundness and apoptosis features reached significance in univariate analysis, but not in multivariate analysis. One aCTC/7.5 ml of blood was found in 7 of 204 healthy controls and 9 of 694 benign tumors. In one patient with benign tumor 2 and another 9 aCTC were detected.

**Significance of the study:**

CTC can be identified and morphological features extracted by an algorithm on images stored by the CellSearch system and strongly correlate with clinical outcome in metastatic breast, colorectal and prostate cancer.

## Introduction

Case studies showing the presence of tumor cells in blood of cancer patients have been reported for more than a century [1]–[4]. These circulating tumor cells (CTC) are extremely rare and technology to reliably enumerate CTC has only become available in recent years [5]. Studies conducted with the validated CellSearch system established the relation between the presence of CTC and poor outcome [6]–[10]. The ability to assess the presence of treatment targets on CTC demonstrates the potential for a real time liquid biopsy and has certainly spurred the interest in CTC [11]–[17].

The recent emergence of different technologies to identify CTC has been accompanied with a large range of CTC definitions urging the need for standardization [5], [18]–[31]. Although different CTC characteristics may indeed relate to clinical outcome [32], it is of utmost importance that counting and characterization of CTC can be done accurately, reproducibly, and is validated in controlled clinical studies. Studies have shown that even in the FDA cleared CellSearch system intra-reviewer, inter-reviewer, and inter-laboratory variability is substantial [33], [34]. Recently, we developed a CTC detection algorithm that counts CTC in images recorded by the CellSearch system [35]. This algorithm was not developed to copy human reviewers that assign events as CTC, but it used survival data of metastatic prostate cancer patients to arrive at a definition that optimally stratified the patients into groups with favorable and unfavorable survival [35]. This algorithm eliminates reviewer variability, is fast and decreases the cost of the CTC assay by elimination of the time a reviewer spends on reviewing the images. Furthermore, it delivers quantitative information about the objects it counts as CTC. CTC are morphologically very heterogeneous ^5^ and in case studies in breast and colorectal cancer it was found that breast cancer CTC are somewhat rounder than cells from colorectal samples [36] In this study, we validated the CTC algorithm on images of patients with metastatic colorectal and breast cancer and evaluated the correlation of quantitative morphological parameters with clinical outcome of patients with metastatic prostate, colorectal and breast cancer before and after initiation of a new line of therapy.

## Materials and Methods

### Patients

Blood samples enriched for CTC of patients before they received treatment (baseline) and at first follow-up after initiation of a new line of therapy from prospective multicenter CTC studies were used in this study. For metastatic breast cancer, fluorescence images of 283 blood samples processed on the CellSearch system from 179 patients could be imported from the IMMC-01 study and 482 image sets from 248 patients from the IC2006-04 study [7], [37]. For metastatic colorectal cancer, CellSearch images of 1691 image sets from 507 patients could be imported from the CAIRO-2 and IMMC-06 studies [6], [38]. For castration resistant prostate cancer (CRPC), CellSearch images of 370 image sets from 185 patients could be imported from the IMMC-38 study and 189 image sets from 100 patients could be imported from the Abiraterone study [8], [39], [40]. From the IMMC-26 study 317 image sets from 93 patients with benign colorectal disease and 200 image sets from 61 patients with benign breast disease were imported [41], [42]. In addition, 204 image sets from healthy controls from the IMMC-01 and IMMC-06 study were included in this study. In Table S1 the detailed characteristics of the patients enrolled in these studies are shown. For these studies all patients and healthy volunteers provided written informed consent. The studies were approved by the ethics commissions and institutional review boards of Institut Curie Paris, Royal Marsden, London, Radboud Nijmegen, Medisch Spectrum Twente and centers participating in the original studies [6], [7], [8], [37], [38], [39], [40], [41], [42].

### Manual Enumeration of Circulating Tumor Cells

The CellSearch system (Veridex LLC, Huntingdon Valley, PA, USA) was used to enumerate CTC. The system consists of a CellTracks Autoprep® for sample preparation and a CellTracks Analyzer II® for sample analysis [5], [43] The CellTracks Autoprep immuno-magnetically enriches epithelial cells from 7.5 ml of blood targeting the Epithelial Cell Adhesion Molecule (EpCAM). The enriched sample is labeled with phycoerythrin-conjugated (PE) antibodies directed against cytokeratins (CK) 8, 18 and 19, an allophycocyanin-conjugated (APC) antibody against CD45 and the nuclear dye 4′,6-diamidino-2-phenylindole (DAPI). After preparation the enriched sample is transferred to a cartridge contained in a CellTracks MagNest. All ferrofluid labeled objects are pulled towards the imaging surface of the cartridge, which is placed on the CellTracks Analyzer II for image acquisition. The analyzer is a four-color semi-automated fluorescence microscope that captures digital images in four different fluorescent channels using a 10×/0.45 NA objective and a charge-coupled device camera with 6.7×6.7 µm^2^ sized pixels. For each cartridge, 144–180 4-layer tiff images of DAPI, FITC, PE, APC are saved per patient. After imaging, the system preselects objects that are positive for DNA and CK and shows the images of all fluorescence channels together with a DNA/CK overlay of all selected objects in a gallery. Finally, a trained operator selects objects from the gallery as manual CTC (mCTC) if they are DNA+CK+, CD45-, have a cell-like morphology, and are larger than 4×4 µm^2^.

### Automated Enumeration of Circulating Tumor Cells

The recorded image sets from the patients were copied to central hard drives and analyzed by an algorithm developed in Matlab 2009a, using the DipImage toolbox (Delft University, Delft, The Netherlands). The algorithm was optimized using survival of 185 castration resistant prostate cancer patients as a training parameter and is described in more detail elsewhere [35]. In brief the optimal CTC definition was selected based on 1. High Cox Hazard ratio for borh baseline and follow-up samples; 2 Higher HR for folllow-up than baseline samples; 3 Low relative and absolute number of CTC in 68 control samples. The algorithm was then applied on 144–180 images from each patient. First objects in the CK-PE channel are selected by dynamic thresholding and their outline is saved [44]. Next, the outline of each object is used to perform measurements in the DNA-DAPI, CK-PE, and CD45-APC channels. Finally, the algorithm, optimized using survival data from metastatic prostate cancer patients, selects automated CTC (aCTC). The inclusion criteria for an aCTC were defined as a CK-PE standard deviation higher than 50 counts, a DNA-DAPI peak value of at least 170 counts, a CD45-APC peak value less than 60 counts and a size within 75–500 pixels (35–225 µm^2^). However, the upper aCTC size limit was increased to 2000 pixels (∼900 µm^2^) after the observation that aCTC sizes in metastatic breast cancer are larger [45]. For every patient image set, the included objects were added to arrive at a final aCTC count per patient. To compare the overall aCTC and mCTC enumeration, a frequency plot was created using the data from the three patient groups, benign tumors and healthy controls.

### Additional Morphological Measurements on aCTC

We performed a number of measurements on the aCTC, including cell roundness, nuclear to cytokeratin (NCK) ratio, the presence of cell clusters, and the number of speckles within the cytoplasm. Cell roundness was defined as the ratio of the signal perimeter squared divided by 4π times the signal area (termed P2A in the literature). Thus, roundness equals one for a perfect circle. The NCK-ratio was defined as the area of the DNA-DAPI signal, divided by the area of the CK-PE signal. For this parameter, an extra segmentation step for DNA-DAPI was performed to find DNA-DAPI+ objects larger than 10 µm^2^ (22 pixels, the smallest DNA-DAPI nucleus area). If multiple DNA-DAPI+ objects were found close to the CK signal, the center-of-mass distances between the CK and DNA signals were determined. The object with (partial) overlap between CK and DNA and the smallest distance (no more than 17 µm, or half the size limit) was selected to determine the NCK-ratio. Clusters of cells were defined as the number of DNA-DAPI objects larger than 10 µm^2^ and located within each CK-PE signal. Finally, speckles within the CK-PE signals are indicative of damaged or apoptotic CTC [46]. The number of dot-like structures within the CK-PE signal was counted and used as a measure of apoptosis. Dot-like structures were counted by an algorithm developed for the detection of fluorescence in situ hybridization probes within a cell nucleus [47].

### Identification and Morphological Measurements of Leukocytes

Due to a-specific binding of EpCAM-ferrofluid particles during the CTC enrichment, leukocytes are carried over and are present at the analysis surface of the sample cartridge. Leukocytes are identified by their DAPI staining, expression of CD45 and lack of cytokeratin expression. We applied the triangle threshold method to determine the outline of leukocytes in the CD45-APC channel [44]. Leukocytes were defined as objects that had CK-PE peak value lower than 100 counts, DNA-DAPI peak value of at least 100 counts, CD45-APC peak value of at least 100 counts and a size range between 50 and 2000 pixels (21–900 µm^2^). Morphological parameters were extracted as described for aCTC.

### Statistical Analysis

SPSS 16 (IBM, New York, USA) was used for survival analysis. Least-squares linear regression analysis was performed to compare aCTC and mCTC, baseline and follow-up samples, and to compare different studies of the same type of cancer. Kaplan-Meier analysis was performed by dichotomizing the patient population on the clinically used cut-off value of five CTC for breast and prostate cancer, and three CTC for colorectal cancer. Beyond these cut-offs, other cut-off values of 1, 5, 10, and 100 for aCTC or mCTC were tested to dichotomize the patient population per study for univariate Cox hazard regression. Log-rank tests were used to compare survival between groups. Cox hazard ratios (HRs) with 95% confidence intervals (CI) were determined and collected in a table. Survival was defined as the time between baseline sample and death from any cause.

To determine whether the morphological parameters determined on aCTC provide additional information that is relevant for prognosis, multivariate Cox proportional hazards regression was performed (forward conditional, with *p_in_* 0.10 and *p_out_* 0.20), controlling for cancer type (colorectal, prostate, breast), with all morphological parameters and the aCTC count as continuous variables. The number of aCTC was ^10^log transformed before inclusion in the regression [48], [49]. Because the morphological parameters could not be determined on patients without aCTC, only patients with at least 1 aCTC were included. For parameters that are determined for each individual aCTC, the median value within a patient was used since this represents the predominant characteristic. The nonparametric Mann-Whitney U-test was used to compare morphological parameters of different types of cancer. The t-test could not be used because the parameters are not normally distributed.

## Results

### Frequency of aCTC Versus mCTC

**Figure 1:**
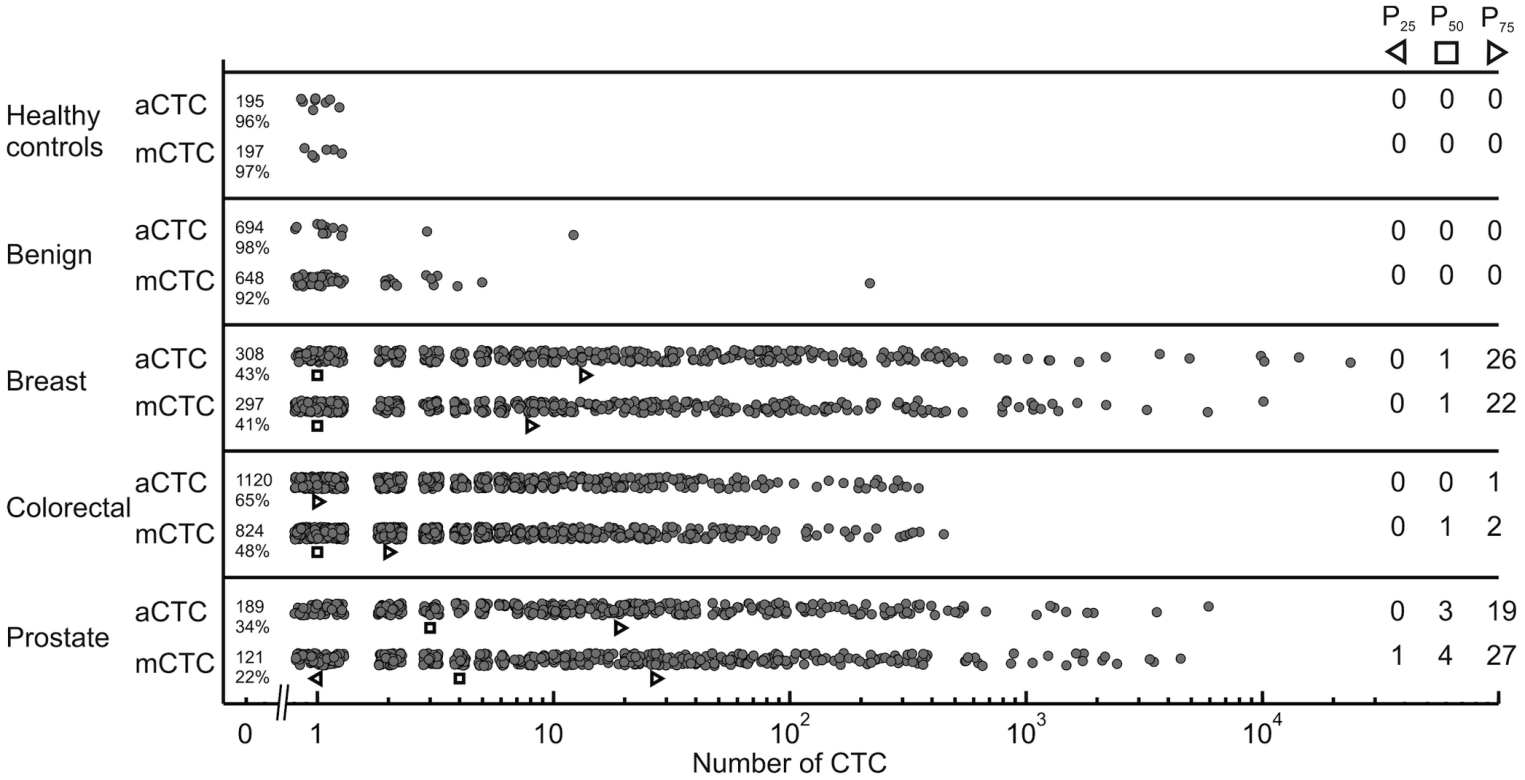
Scatter plot of mCTC and aCTC by cancer type. The frequencies are shown of mCTC and aCTC found in healthy controls, patients with benign disease and patients with metastatic breast, prostate, and colorectal cancer before initiation of a new line of therapy. The dots in the scatter plot are spread for viewing purposes. The 25^th^, 50^th^, and 75^th^ percentile are given by the black markers and in numerical values in the right part of the figure. At the start of each row, the percentage and number of patients that have 0 CTC are shown.

**Figure 2:**
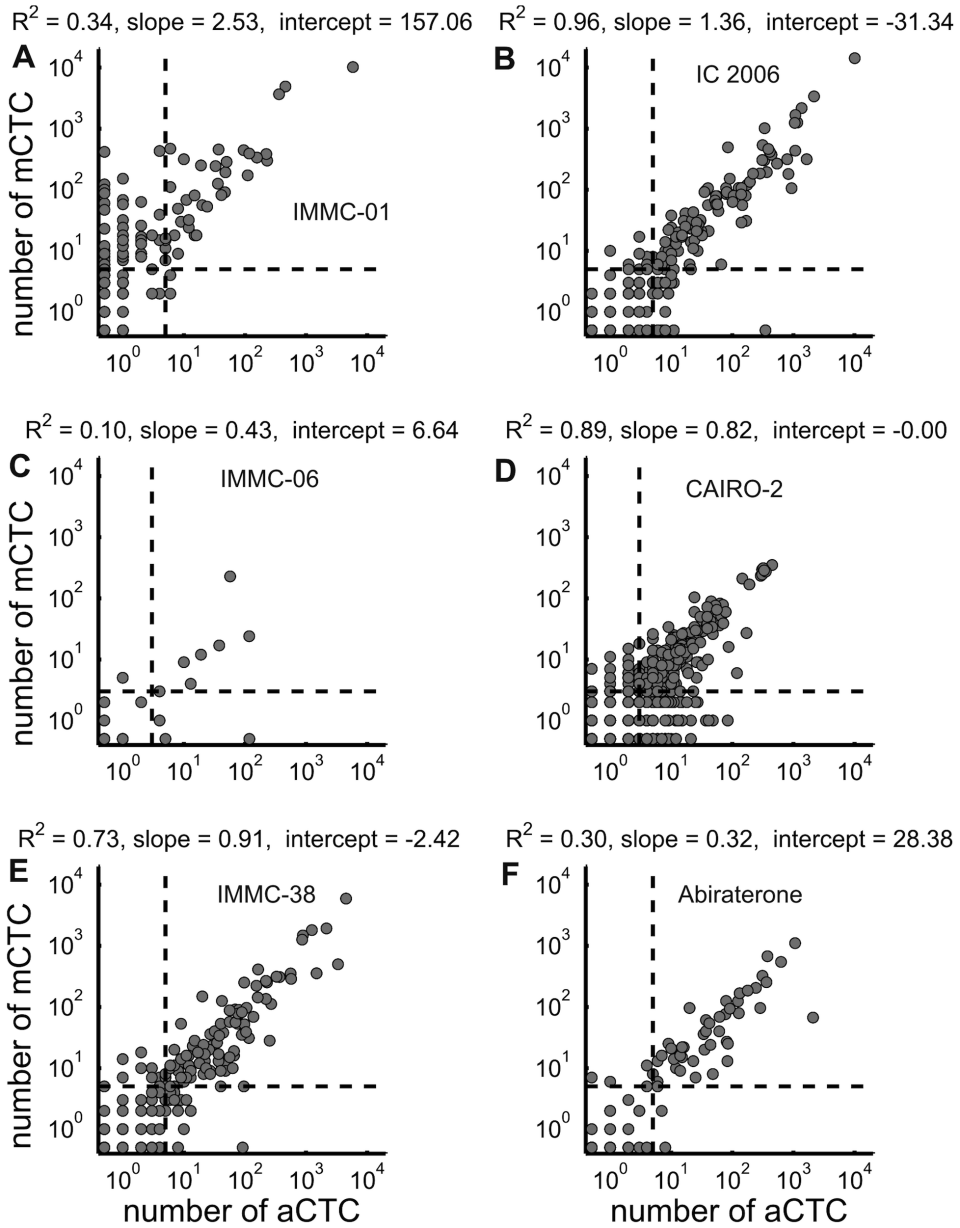
Scatter plot of the number of aCTC versus mCTC for every study. Horizontal and vertical dashed lines were drawn to show clinically used cut-offs, which are ≥5 for breast and prostate, and ≥3 for colorectal cancer. At the top of each graph, the linear regression coefficients are shown.

aCTC and mCTC were enumerated in healthy controls, patients with benign disease and patients with metastatic colorectal, prostate and breast cancer before initiation of therapy. The frequency distribution of the CTC is shown in figure 1 and the 25^th^, 50^th^, and 75^th^ percentiles are provided at the right hand side of the figure. Figure 2 shows the scatter plot of aCTC versus mCTC per study, as well as the linear regression coefficients. Detailed quantitative information about counted ranges, median, mean, SD, and linear regression coefficients are presented in Table S2. In summary, breast CTC counts ranged from 0–10111 aCTC (median 2/0 for baseline/follow-up) and 0–23618 mCTC (median 3/0 for baseline/follow-up); colorectal CTC counts from 0–448 (median 1/0) and 0–351 mCTC (median 0/0); prostate CTC counts ranged from 0–4970 aCTC (median 7/3) and 0–5925 mCTC (median 7/2) per 7.5 ml of blood. Benign patients ranged from 0–218 (median 0) aCTC and 0–12 mCTC (median 0). Finally, healthy controls had up to 1 aCTC and mCTC (both medians 0). Correlation R^2^ between mCTC and aCTC was 0.48 for 725 breast samples, 0.84 for 1729 colorectal samples, and 0.68 for 559 prostate samples.

### Measurements of aCTC Morphological Parameters

**Figure 3:**
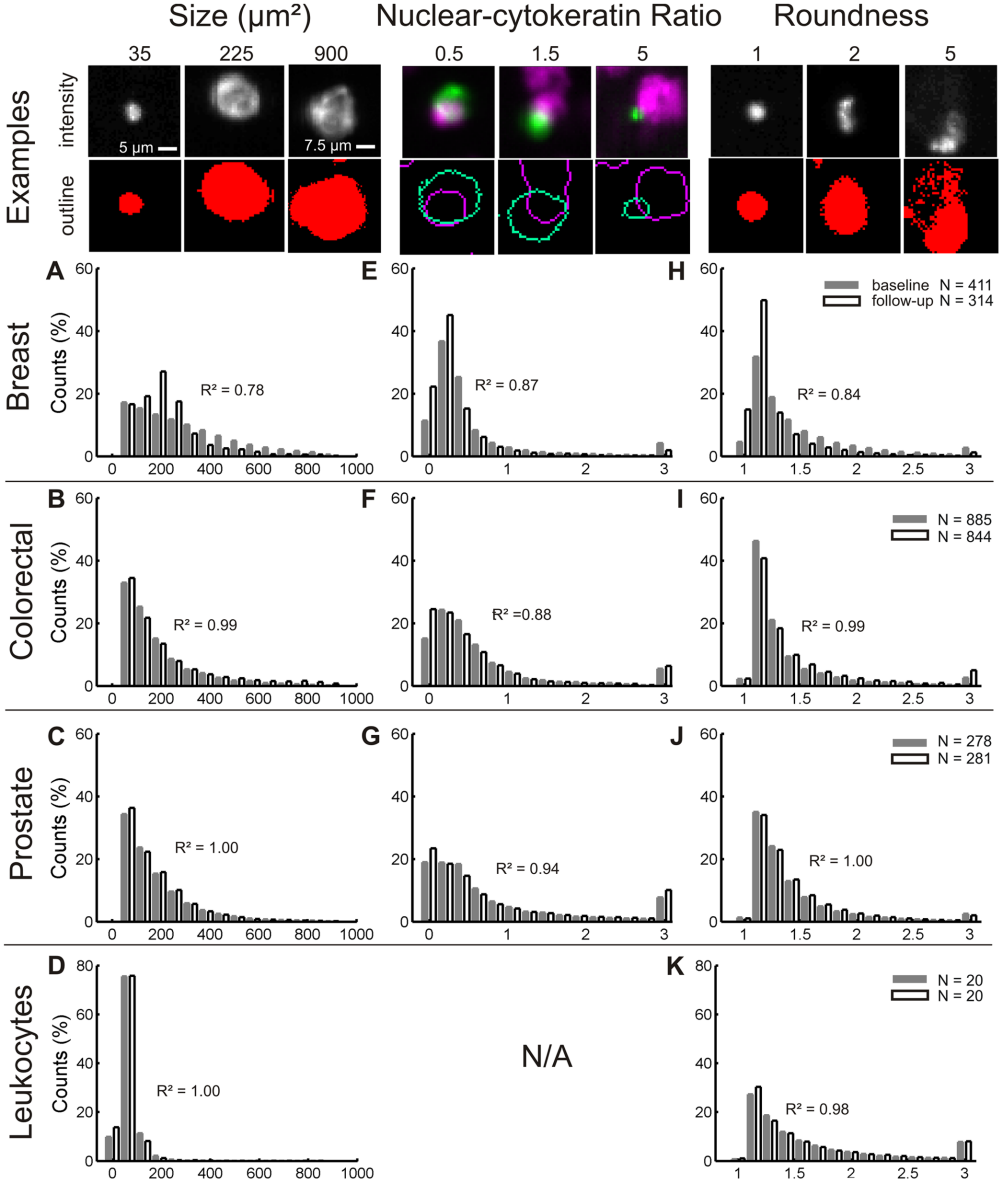
Histograms of morphological parameters of aCTC by cancer type. Breast (panel A, E, H), colorectal (panel B, F, I), and prostate samples (panel C, G, J) are shown next to morphological parameters of leukocytes (panel D, K). Baseline samples (black bars) are shown next to first follow-up samples (white bars). Coefficients of determination of a linear regression between the time points are shown next to the bars. Examples of morphological parameter values of aCTC are given at the top of the figure, as well as examples of the outlines after segmentation. The objects seem smaller in the images than in the outlines. This is due to scaling of the image for print, the outlines show the area of the object that is distinguishable from noise, and thus represents the true size of the object. The 5 µm scale bar applies to all images if not stated differently. N/A: not applicable.

Recording of the outline of aCTC permits the extraction and quantification of morphological parameters from the identified aCTC. The distribution of the size, NCK ratio and roundness of aCTC in blood of patients with metastatic breast, colorectal, and prostate cancer before initiation of therapy and at first follow up after initiation of therapy is shown in figure 3. Examples for these three morphological parameters are shown at the top of the figure, with histograms for CTC from colorectal, breast and prostate cancer below each parameter. The aCTC size (µm^2^) distribution for colorectal, breast and prostate cancer is shown in panels A, B and C respectively. The NCK-ratio (range 0.5 to 5) for breast, colorectal, and prostate cancer CTC is shown in panels E, F and G respectively. aCTC appear in a large range of shapes. To illustrate this diversity in CTC shape, a measure of roundness (range 1 to 5) for colorectal, breast and prostate cancer CTC is shown in panels H, I, and J respectively. For comparison the size and roundness properties of leukocytes are shown in figure 3, panels D and K. Leukocytes have a narrow size distribution, as can be seen in panel D. The NCK-ratio is not shown as leukocytes do not express cytokeratin. R^2^ correlation between measurements before and after initiation of therapy is provided in each panel. aCTC morphological parameters were quantified by mean and standard deviation and are provided in table 1. To test whether the morphological parameters from table 1 were significantly different between cancer types, Mann-Whitney U-test p-values were derived using all image sets of each type of cancer. Derived p-values for median size of aCTC between colorectal and prostate was 0.90 and 0.35 for median NCK-ratio between breast and colorectal. The percentage apoptotic cells between breast and prostate had *p* = 0.18. For median roundness of aCTC *p*-values were 0.27, 0.008, and 0.15 for comparison between breast-colorectal, breast-prostate, and colorectal-prostate, respectively. For all other morphological parameters from table 1 *p* was less than 0.001 between all types of cancer.

**Table 1:** Size, roundness and nuclear to cytokeratin ratio of CTC in breast, colorectal cancer and leukocytes present in the CTC enriched samples.

	Time point	Breast		Colorectal		Prostate		Leukocytes	
		mean	(SD)	mean	(SD)	mean	(SD)	mean	(SD)
Size (µm^2^)	BL	290	(200)	186	(153)	180	(145)	71	(44)
	FU1	220	(142)	209	(192)	174	(141)	66	(47)
Roundness	BL	1.5	(0.6)	1.5	(1.4)	1.5	(0.9)	1.8	(1.2)
	FU1	1.1	(0.7)	1.3	(1.6)	1.3	(0.9)	1.4	(1.1)
NCK ratio	BL	0.8	(2.8)	1.0	(3.5)	1.0	(2.1)	NA	
	FU1	0.5	(2.0)	1.1	(4.9)	1.3	(4.3)	NA	

SD = standard deviation; BL = baseline; FU1 = first follow-up; Roundness of 1 indicates perfectly round; NCK = nucleus to cytokeratin ratio 1 indicates equal areas. NA = not applicable.

The size of the objects can be influenced by the presence of multiple CTC representing CTC clusters. These clusters are counted as one CTC in both the manual identification as well as the automated identification of CTC. The presence of aCTC clusters was estimated by the number of nuclei that could be detected within the CK-PE area. Percentages of clustered cells per patient are shown in table 2 for patients with ≥5 aCTC for image sets of both baseline and follow-up. None of the patients showed a significant rise or drop (outside 95% CI) in the number of clusters after initiation of therapy. The presence of speckles of cytokeratin is thought to be associated with CTC undergoing apoptosis. As a measure of these speckled aCTC the percentage of all aCTC that have 3 or more dot-like structures in their cytokeratin signal is shown in table 2 for both baseline and follow-up samples**.** None of the patients had a significant rise or drop in the number of speckled aCTC between baseline and first follow-up measurement.

**Table 2:** Clusters and speckled aCTC at baseline and follow-up samples from breast, colorectal and prostate cancer patients with ≥5 aCTC.

	% Clustered/Total aCTC						% Speckled/Total aCTC					
	Baseline			First follow-up			Baseline		First follow-up			
	mean	med	range	mean	med	range	mean	med	range	mean	med	range
Breast (n = 111)	3.8	2.2	0–30	3.2	0.0	0–20	3.5	2.1	0–12	5.5	2.9	0–22
Colorectal (n = 100)	5.2	0.0	0–40	8.6	0.0	0–40	3.2	0.0	0–27	6.0	0.0	0–40
Prostate (n = 49)	5.1	2.9	0–40	4.7	3.5	0–36	3.4	2.1	0–25	3.3	0.9	0–25
Leukocytes (n = 20)	0.0	0.0	0–0	0.0	0.0	0–0	0.0	0.0	0–0	0.0	0.0	0–0

Cluster of aCTC defined as two or more DNA-DAPI objects within their CK-PE outline Speckled aCTC defined as more than two dot-like structures in their CK-PE outline.

### mCTC, aCTC and Morphological Parameters Versus Survival in Metastatic Breast, Colorectal and Prostate Cancer

**Figure 4:**
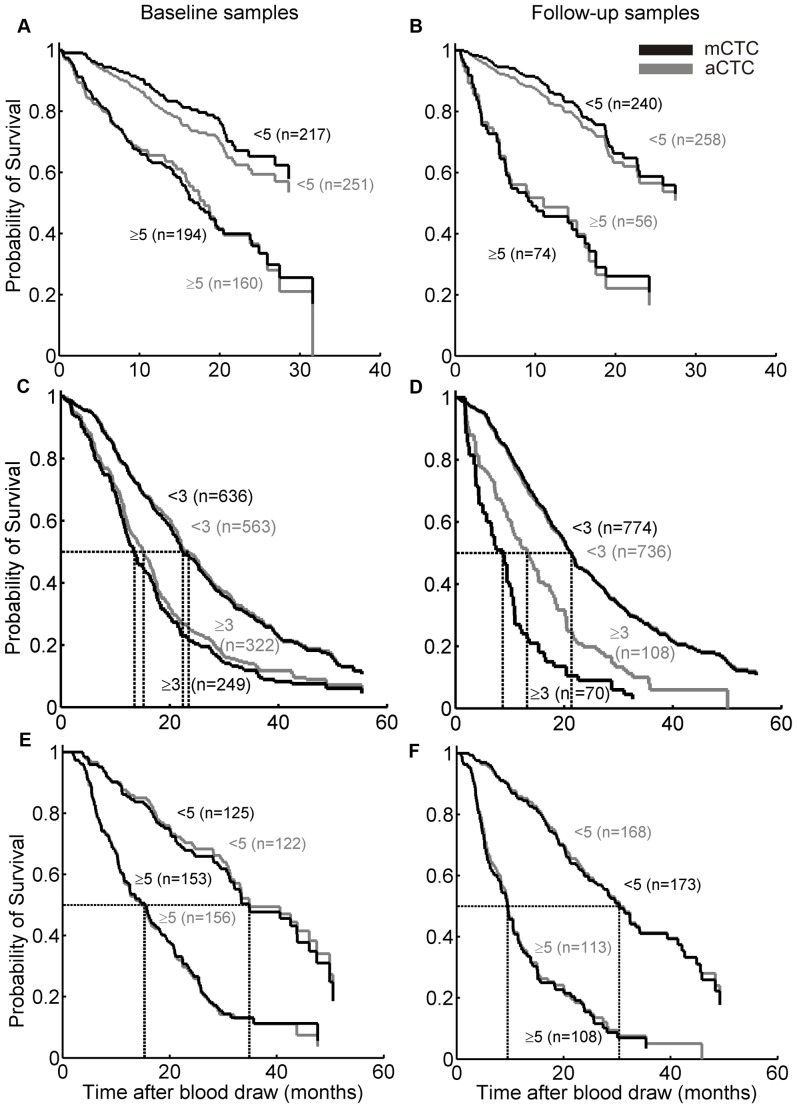
Kaplan-Meier plots of baseline and follow-up samples by cancer type, and mCTC, or aCTC. Metastatic breast (panel A and B), colorectal (panel C and D), and prostate cancer patients (panel E and F) are shown with favorable and unfavorable mCTC (black lines) and aCTC definition (grey lines). For every type of cancer, patients were pooled from the two studies of each type as shown in Table S1.

To illustrate the relationship between survival and CTC, Kaplan Meier plots were generated dividing the patients in favorable and unfavorable groups based on a threshold of 3 CTC for metastatic colorectal cancer and 5 CTC for metastatic prostate and breast cancer. The Kaplan-Meier plots shown in figure 4 are for aCTC and mCTC before initiation of therapy (panels A, C, E) and at first follow-up after initiation of therapy (panels B, C, F). The relationship between aCTC number and overall survival in metastatic breast colorectal, and prostate cancer is shown in table 3. To investigate the impact of aCTC morphological parameters on survival, univariate and multivariate analysis was performed using the measured morphological parameters as covariates next to the number of aCTC or mCTC. HRs were calculated for each covariate as a continuous variable. Table 4 shows the HRs, derived p-values, and chi-squared.

**Table 3:** Relation between aCTC number and overall survival in metastatic colorectal, breast and prostate cancer.

	aCTC			mCTC		
cut-off	n at risk	% at risk	HR† (95% CI‡)	n at risk	% at risk	HR (95% CI)
**Breast** ≥1	**BL** 270	66	1.5 (1.1–2.2)	279	68	2.8 (1.8–4.2)
≥5	160	39	2.3 (1.7–3.3)	194	47	3.0 (2.1–4.2)
≥10	120	29	2.5 (1.8–3.5)	151	37	3.1 (2.2–4.3)
≥100	43	10	2.3 (1.4–3.6)	54	13	2.7 (1.8–4.1)
	**FU1**					
≥1	151	48	1.8 (1.2–2.6)	139	44	3.3 (2.2–5.0)
≥5	56	18	3.7 (2.5–5.7)	74	24	4.2 (2.9–6.3)
≥10	40	13	3.7 (2.3–5.9)	55	18	3.9 (2.6–5.9)
≥100	6	2	8.3 (3.0–23.5)	14	4	4.1 (2.1–7.7)
**Colorectal**	**BL**					
≥1	565	64	1.7 (1.4–2.0)	437	49	1.8 (1.5–2.1)
≥3	322	36	1.7 (1.5–2.0)	249	28	1.9 (1.6–2.2)
≥10	139	16	1.9 (1.6–2.3)	114	13	2.3 (1.8–2.8)
≥100	13	1	3.9 (2.2–6.9)	11	1	6.1 (3.3–11.3)
	**FU1**					
≥1	340	40	1.5 (1.2–1.7)	172	20	2.1 (1.8–2.6)
≥3	108	13	2.0 (1.6–2.5)	70	8	3.8 (2.7–4.7)
≥10	27	3	4.6 (3.0–6.9)	19	2	13.3 (8.1–21.7)
≥100	4	0.5	24.1 (8.5–68.3)	4	0.5	24.1 (8.5–68.3)
**Prostate**	**BL**					
≥1	231	83	2.9 (1.8–4.6)	205	74	2.7 (1.9–3.9)
≥5	156	56	3.4 (2.5–4.8)	153	55	3.1 (2.3–4.3)
≥10	123	44	3.0 (2.2–4.0)	118	42	3.3 (2.4–4.5)
≥100	35	13	3.7 (2.5–5.6)	31	11	5.5 (3.5–8.4)
	**FU1**					
≥1	207	74	2.3 (1.6–3.4)	165	59	3.9 (2.8–5.5)
≥5	113	40	4.1 (3.0–5.5)	108	38	4.2 (3.1–5.8)
≥10	95	34	3.9 (2.8–5.3)	83	30	4.5 (3.3–6.2)
≥100	29	10	5.2 (3.4–8.1)	23	8	4.0 (2.4–6.6)
						

† Cox hazard ratio,

‡ confidence interval.

**Table 4:** Univariate analysis of variables for all follow-up samples used in this study.

Variable	HR	(CI 95%)	p	Chi-square
10log(mCTC)	1.5	1.4–1.5	<0.001	203.4
10log(aCTC)	1.7	1.5–1.8	<0.001	178.1
median roundness	0.86	0.79–0.94	0.001	9.6
median size (µm)	0.98	0.96–0.99	0.002	9.2
% speckled	0.99	0.99–0.99	0.016	5.8
% clusters	0.99	0.99–1.00	0.137	2.2
median NCK ratio	0.98	0.94–1.00	0.299	1.1

Next, all variables of aCTC were entered in a multivariate Cox regression analysis. mCTC were left out of this analysis, as their quantitative parameters are unknown. Hazard models were calculated using all first follow-up image sets for all cancer types together and for each type separately. When all image sets were combined, we controlled for differences in overall survival due to cancer type. Results are shown in table 5. The optimal model used the number of aCTC, and their median roundness per patient and had a Chi-square of 181 with 3 degrees of freedom (with the third degree the control variable for colorectal cancer). The model shows that more objects impose a bigger threat and that objects with a roundness close to one may be more dangerous to patients than objects that are elongated. When including only samples from one type of cancer, the median size of the aCTC, and the median NCK-ratio of the aCTC had a significant influence on survival in breast cancer samples. HRs smaller than one show that in breast cancer small objects represent greater hazard than large objects. Furthermore, objects with small DAPI signals with respect to their CK signal, i.e. a low NCK-ratio, are more dangerous for patients than cells with high NCK-ratio. Other morphological parameters did not improve the multivariate models.

**Table 5:** Multivariate analyses of aCTC variables on all first follow-up samples, and for each type of cancer.

Variable	HR	CI 95%	p	Chi-square
**All**				
log(aCTC)	2.5	2.2–2.9	0.001	180.8
median roundness	0.9	0.8–0.9	0.04	
colon cancer	1.8	1.4–2.2	<0.01	
**Breast**				
log(aCTC)	2.9	2.0–4.0	<0.001	36.7
median size (µm)	0.9	0.9–0.9	0.018	
median NCK-ratio	0.5	0.3–0.9	0.026	
**Colon**				
log(aCTC)	2.7	2.0–3.5	<0.001	50.1
**Prostate**				
log(aCTC)	2.6	1.9–3.5	<0.001	42.3

Chi-square is given for comparison of the whole model with the null hypothesis, degrees of freedom is equal to the number of variables shown. Multivariate analysis was performed by forward stepwise regression.

## Discussion

Recently, we showed that counting CTC by an automated algorithm is preferable to manual counting by a trained reviewer [35]. The algorithm performed similar to the human reviewer in terms of prognostic value: similar HRs were found. In addition, the algorithm takes no operator time and has 0% variability, against an inter-laboratory variability of 4% to 31% for mCTC (median 14%) reported previously by another group [33]. The aCTC algorithm was optimized to obtain the best separation in survival risk and not to obtain the best correlation with mCTC. The training set used image sets from a prospective metastatic prostate cancer study and was validated on an independent data set of prostate cancer patients included in the Abiraterone phase I/II trials [39], [40]. The aCTC algorithm with the highest prognostic value included objects with DNA and CK, without CD45, and which have a size between 34 µm^2^ and 224 µm^2^.

Here we applied the aCTC algorithm to image sets of patients with benign and malignant breast and colorectal cancer, as well as to image sets of healthy controls. The upper size limit was increased from 224 µm^2^ to 900 µm^2^, concordant with a recent study showing a larger size of CTC in breast cancer [45]. This change resulted in an aCTC increase by a factor of 1.3 for prostate cancer with less than 15% effect on the HR. The increase in the number of aCTC was a factor 1.4 for colorectal image sets and 1.9 for breast cancer image sets, and thus indicative for the larger average size of CTC in breast cancer. The frequency distribution of CTC illustrated in figure 1 shows that the aCTC and mCTC counts do not differ greatly. Separation of the results into the different studies however showed a clear discrepancy between the counts obtained with samples from the IMMC-01 breast cancer study as compared to all other studies (figure 2b). The cause of this discrepancy was traced back to the CD45 staining of objects, as the CD45 staining in the IMMC-01 study was much higher as compared to the IC2006–04 study. This resulted in the rejection of many objects in the IMMC-01 study. Lowering of the CD45 threshold eliminated the discrepancy between the number of aCTC in the IMMC-01 and other studies (data not shown). IMMC-01 was the first of all studies in this report, potential causes for the higher CD45-APC signal in IMMC-01 are a higher concentration of the CD45-APC fluorochrome, differences in sample preparation or differences in filter cubes giving rise to more crosstalk of CK-PE signal into the CD45-APC channel. Most image sets of IMMC-01, 06 and 38 were scanned on the CellSpotter Analyzer instead of the CellTracks Analyzer II used for the other studies, therefore differences between CellTracks and CellSpotter do not offer an explanation for the differences in CD45-APC signal. The IMMC-01 study was the first large clinical trial run with the CellSearch system and during this study blood samples were not automatically processed by the CellSearch Autoprep system. Kaplan-Meier analysis on the IC2006–04 study alone showed that the aCTC and mCTC had the same prognostic value. Therefore, future breast cancer blood samples, processed using current CellSearch equipment, can be accurately analysed with the algorithm. As different microscopes (CellSpotter Analyzer & CellTracks Analyzer II) were used in this study one might assume that other microscopes can be used for the analysis of the images with this algorithm. One however has to keep in mind that the same objective (10X, NA 0,45) was used. For successful application of another imaging system it is of great importance that the size in the sample corresponding to a pixel, and the typical intensity of a tumor cell will need to be matched. The control samples did not contain more than one aCTC or mCTC, showing that these definitions have very few false positives. The aCTC frequencies detected in patients with benign tumors were slightly less as compared to the mCTC frequencies, as were the numbers found in cancer patient blood samples. From Figure 4 and table 3 it is evident that identification of CTC by manual review and the automated algorithm performs equally well in discriminating between patients with favourable and unfavourable outcome. Except for the follow-up image sets of the colorectal cancer patients. The latter may be contributed to the low incidence of CTC for this group of patients.

Comparison of aCTC morphological parameters between cancer types shows a larger size, a relatively large nucleus to cytokeratin ratio and rounder CTC in breast cancer as compared to prostate and colorectal cancer. The distribution of the size of aCTC is exponential in CTC of both prostate and colorectal cancer, which is cut off at the lower end by the CTC algorithm, in contrast with leukocytes that display a more bell-shaped size distribution. These distributions shows that a wide range of CTC and CTC fragments are present in the blood of patients, as was reported earlier [46]. It was found in earlier work that the number of tumor micro particles (TMPs) is also prognostic for survival [32], it is therefore interesting to include these TMPs in the analysis, as they are present in abundant numbers. Especially in colorectal cancer, TMPs might reduce the large Poisson error that is currently present by using a clinical cut-off value of three mCTC. Clusters and speckled cells were found among aCTC, but not among leukocytes. A few exceptional colorectal samples contained a large number of apoptotic cells and clusters, but the presence of these cells or clusters had no detectable impact on survival. Breast cancer aCTC showed the highest number of speckled cells, while prostate cancer aCTC had more clusters, although the differences between cancer types are small. No significant rise or drop in the number of clusters or apoptotic cells was found between baseline and follow-up samples in any patient.

We performed continuous univariate and multivariate survival analysis to investigate the influence of aCTC and its morphological parameters on survival. CTC measurements at first-follow up after treatment were used for this analysis as the CTC “surviving” this treatment more strongly related to survival and might contain typical morphological features. Median roundness was significant (*p*<0.05) when we included all cancer types in the multivariate analysis, with rounder cells predicting poorer survival. When we performed multivariate analysis by cancer type, small cells with small nuclei compared to the cytoplasm were significantly (*p*<0.05) associated with poor survival only for breast cancer patients. Currently, we have no biological explanation for this phenomenon. However, research focused on separating normal blood cells from CTC on the basis of size should consider this observation. The association of rounder and/or smaller cells with poorer survival suggest that these maybe features of cancer stem cells, similar to the observations made for hematopoietic stem cells, which are clearly smaller and rounder as compared with the other hematopoietic progenitor cells [50]. If this is indeed the case size dependent methods for CTC enrichment will not be able to detect these putative cancer stem cells.

In conclusion, we have validated a CTC automated algorithm, which was trained on samples from metastatic prostate cancer patients, on other types of cancer samples and samples from patients with benign tumors and healthy controls. We show that differences exist in physical characteristics between CTC from different origin, although their impact on survival is limited. Standardized identification and counting of CTC is imperative to move the field forward and will enable the molecular characterization of those objects/cells that are indeed CTC.

## Supporting Information

Table S1 **Patient characteristics.**
(DOCX)

Table S2. (DOCX)
