# Supplementary material for: Circulating Tumor Cells Count and Morphological Features in Breast, Colorectal and Prostate Cancer
Source: PLoS One. 2013 Jun 27;8(6):e67148. doi: 10.1371/journal.pone.0067148 (PMC3695007; doi:10.1371/journal.pone.0067148)
Supplement: Table S1 — Patient characteristics. (DOCX) [file pone.0067148.s001.docx]

**Supplemental Table S1**

Patient characteristics

| Study | **IMMC-01** | **IC2006-04** | **Cairo-2** | **IMMC-06** | **IMMC-38** | **Abirat-erone** |
| --- | --- | --- | --- | --- | --- | --- |
| Cancer | Breast | Breast | Colorectal | Colorectal | Prostate | Prostate |
| N patients | 179 | 248 | 417 | 34 | 185 | 100 |
| N samples | 283 | 442 | 1690 | 39 | 370 | 189 |
| N censored | 75 | 193 | 180 | 10 | 67 | 26 |
| Survival† (months) | 15.1 | 13.1 | 18.7 | 23.4 | 16.9 | 22.2 |
|  | (0.4 - 48.6) | (0.2-33.3) | (0-60.8) | (0.8 - 39.1) | (1.9 - 38.7) | (0.9-53.2) |
| Follow-up censored†† (months) | 20.7 | 14.2 | 36.7 | 31.8 | 25.9 | 40.8 |
|  | (1.3 - 48.6) | (1.4-33.3) | (0-60.8) | (22.8 - 39.1) | (2.4 - 38.7) | (9.2-53.2) |
| Therapy line‡ | 43/15/42% | 100/0/0% | 100/0/0% | 62/29/9 | 67/16/17% | 0/20/80% |
| Age† | 58 (27 - 86) | 57 (28-84) | 63 (27-84) | 65 (45 - 83) | 69 (49 -92) | 70 (50-84) |

† Median values shown with range in parentheses, †† median duration of follow-up for censored patients with range in parentheses ‡ % of patients with 1^st^, 2^nd^, 3+ Line of chemotherapy (% 1^st^/ % 2^nd^/ % 3+).
