# Supplementary material for: Circulating Tumor Cells Count and Morphological Features in Breast, Colorectal and Prostate Cancer
Source: PLoS One. 2013 Jun 27;8(6):e67148. doi: 10.1371/journal.pone.0067148 (PMC3695007; doi:10.1371/journal.pone.0067148)
Supplement: Table S2. — (DOCX) [file pone.0067148.s002.docx]

**Supplemental Table S2**

| **Study** | **Timepoint** | **range aCTC** | **range mCTC** | **R^2^ aCTC-mCTC** |
| --- | --- | --- | --- | --- |
|  | **(N)** | **(median/mean/SD)** | **(median/mean/SD)** | **(slope/intercept)** |
| IMMC01 | BL* (163) | 0-5868 | 0-23618 | 0.34 |
| (breast) |  | (1/58/472) | (5/305/2058) | (2.53/157.06) |
|  | FU1†(120) | 0-3248 | 0-9864 | 0.99 |
|  |  | (0/36/298) | (1/116/905) | (2.99/7.68) |
| IC2006-04 | BL (247) | 0-10111 | 0-14274 | 0.96 |
| (breast) |  | (3/130/735) | (2/121/932) | (1.36/-31.34) |
|  | FU1 (194) | 0-221 | 0-202 | 0.66 |
|  |  | (1/7/24) | (0/7/24) | (0.80/1.06) |
| IMMC-06 | BL (21) | 0-118 | 0-229 | 0.10 |
| (colorectal) |  | (2/19/36) | (0/15/50) | (0.43/6.64) |
|  | FU1 (18) | 0-69 | 0-10 | <0.001 |
|  |  | (2/10/21) | (0/0.8/2) | (0.002/0.82) |
| CAIRO-2 | BL (864) | 0-448 | 0-351 | 0.89 |
| (colorectal) |  | (1/8/34) | (0/7/29) | (0.82/-0.01) |
|  | FU1 (827) | 0-232 | 0-197 | 0.90 |
|  |  | (0/2/14) | (0/2/12) | (0.82/-0.22) |
| IMMC-38 | BL (185) | 0-4509 | 0-5925 | 0.73 |
| (prostate) |  | (8/114/469) | (7/101/496) | (0.91/-2.42) |
|  | FU1 (185) | 0-1628 | 0-545 | 0.63 |
|  |  | (3/44/152) | (2/30/89) | (0.46/9.50) |
| Abiraterone | BL (93) | 0-2099 | 0-1108 | 0.30 |
| (prostate) |  | (5/77/256) | (6/53/151) | (0.32/28.38) |
|  | FU1 (96) | 0-3476 | 0-3573 | 0.74 |
|  |  | (3/122/493) | (2/74/390) | (0.68/-9.46) |
| Benigns | N/A‡ (707) | 0-218 | 0-12 | N/A |
| (breast, colorectal) |  | (0/0.4/8.2) | (0/0.04/0.5) |  |
| Healthy controls | N/A (204) | 0-1 | 0-1 | N/A |
|  |  | (0/0.03/0.2) | (0/0.03/0.2) |  |

*baseline; †first follow-up, ‡not applicable
